# The effects of transcription factor competition on gene regulation

**DOI:** 10.3389/fgene.2013.00197

**Published:** 2013-10-07

**Authors:** Nicolae Radu Zabet, Boris Adryan

**Affiliations:** ^1^Cambridge Systems Biology Centre, University of CambridgeCambridge, UK; ^2^Department of Genetics, University of CambridgeCambridge, UK

**Keywords:** transcription factors, facilitated diffusion, noise, molecular crowding, roadblocks

## Abstract

Transcription factor (TF) molecules translocate by *facilitated diffusion* (a combination of 3D diffusion around and 1D random walk on the DNA). Despite the attention this mechanism received in the last 40 years, only a few studies investigated the influence of the cellular environment on the facilitated diffusion mechanism and, in particular, the influence of “other” DNA binding proteins competing with the TF molecules for DNA space. Molecular crowding on the DNA is likely to influence the association rate of TFs to their target site and the steady state occupancy of those sites, but it is still not clear how it influences the search in a genome-wide context, when the model includes biologically relevant parameters (such as: TF abundance, TF affinity for DNA and TF dynamics on the DNA). We performed stochastic simulations of TFs performing the *facilitated diffusion* mechanism, and considered various abundances of cognate and non-cognate TFs. We show that, for both obstacles that move on the DNA and obstacles that are fixed on the DNA, changes in search time are not statistically significant in case of biologically relevant crowding levels on the DNA. In the case of non-cognate proteins that slide on the DNA, molecular crowding on the DNA always leads to statistically significant lower levels of occupancy, which may confer a general mechanism to control gene activity levels globally. When the “other” molecules are immobile on the DNA, we found a completely different behavior, namely: the occupancy of the target site is always increased by higher molecular crowding on the DNA. Finally, we show that crowding on the DNA may increase transcriptional noise through increased variability of the occupancy time of the target sites.

## 1. Introduction

Transcription factors (TF) are DNA-binding proteins that regulate gene activity by binding to specific sites on the DNA. Riggs et al. (1970) observed that the association rate of the lac repressor (a bacterial TF) to its target site is much faster than predicted by simple 3D diffusion. It was later proposed that the mechanism by which TF molecules locate their target sites assumes a combination of 3D diffusion and 1D random walk on the DNA, which is often called *facilitated diffusion* (Berg et al., 1981; Halford and Marko, 2004). Their rationale was that the speed-up in target site finding is achieved by reducing the dimensionality of the search process. The existence of facilitated diffusion was proven experimentally both *in vitro* (Kabata et al., 1993) and *in vivo* (Elf et al., 2007).

Following this initial work, a large number of theoretical studies investigated the search process and described the effects that various factors have on the speed at which TFs locate their target sites. With a few exceptions, these studies considered the case of one TF molecule performing the search process on naked DNA, without any competitor species. It is clear that this is an approximation that needs further investigation, because other proteins, including TFs with different specificity, are translocating on the DNA at the same time. In fact, the proportion of inaccessible DNA is high; for example, between 10 and 50% of the *E.coli* DNA is bound by other proteins (which we call “non-cognate”) (Flyvbjerg et al., 2006).

Usually, it is assumed that only one molecule performs the random search (Halford and Marko, 2004; Mirny et al., 2009), but in bacterial cells, TFs usually display 10–100 copies per cell (Wunderlich and Mirny, 2009). Thus, the TF copy number could potentially influence the search time (Foffano et al., 2012) and, consequently, the amount of time the target sites are occupied.

The question that we address in this manuscript is: how does the abundance of TFs and the presence of other molecules on the DNA influence TF target site finding and binding? In particular, we are interested in describing both the mean and the variability (“noise”) of the association rate to a specific target site and of the proportion of time this target site is occupied.

There is a notion that crowding on the DNA can have two opposing effects: (1) reducing the amount of DNA that needs to be “scanned” by covering non-specific sites (Mirny et al., 2009) and (2) increasing the probability that the target site is already covered by non-cognate molecules (Flyvbjerg et al., 2006). In other words, by increasing the abundance of non-cognate molecules, the amount of DNA that needs to be scanned is reduced, but, at the same time, the probability that the target site is occupied by a non-cognate molecule is increased. This suggests that there may be a level of DNA occupancy which optimizes the search speed.

Murugan (2010) proved the existence of an optimal amount of crowding analytically, but their approach contained approximations that could introduce biases in the final results. One of their assumptions was that the sliding length is inversely proportional to the number of molecules bound to the DNA, which is true only if a bound molecule performs just 1D random walks and does not hop or jump, which are commonly accepted modes of TF translocation (Bonnet et al., 2008; Wunderlich and Mirny, 2008). Furthermore, Murugan (2010) disregarded the fact that the non-specific association rate is decreased when the DNA is occupied by other molecules and that the target site can also be occupied by non-cognate molecules. When these aspects are taken into account, Li et al. (2009) showed that the time to locate the target site always increases with increasing amounts of crowding on the DNA. However, aforementioned studies (Flyvbjerg et al., 2006; Li et al., 2009; Murugan, 2010) assumed that the proteins bound to the DNA act as fixed obstacles, i.e., they do not move on the DNA. This approximation needs further analysis, because non-cognate TF molecules will display similar dynamic behavior to the cognate TFs under investigation.

Marcovitz and Levy (2013) addressed the question of the difference between mobile and immobile obstacles and found that, in the case of immobile obstacles, there is a crowding level that minimizes the search time, while, in the case of mobile obstacles, the search time grows monotonically with increasing crowding levels. Their model displayed a higher level of detail (by representing explicitly the 3D structure of the DNA and the 3D diffusion of molecules), which meant that they could only focus on a small system of 100 bp of DNA and obstacles covering 2 bp. While this model might accurately represent an *in vitro* system, the size of the DNA is prone to affect the applicability of the results for *in vivo* systems (where the model has to consider the entire genome); as we proposed in Zabet (2012).

As an important step from these previous studies (that were either restricted to smaller subsystems that are relevant only for *in vitro* studies, or relied on mean field approximations), we address the question of how molecular crowding on the DNA influences the TF search process, and the occupancy of the target site, in the context of a comprehensive model of the facilitated diffusion mechanism (Zabet and Adryan, 2012a,c). In particular, our model considers the entire DNA with multiple DNA binding molecules and is fed with parameters that were estimated from experimental measurements (which leads to biologically relevant representation of the bacterial cells). Using a well-characterized TF and its best known binding site as a model, our results indicate that the average time the *E.coli* lac repressor (lacI) requires to locate the *O*_1_ site is increased with the addition of non-cognate molecules that move on the DNA (supporting the result of Li et al., 2009), while, in the case of fixed roadblocks on the DNA, there seems to be a crowding level that optimizes the mean of the search time; supporting the results of Murugan (2010). Nevertheless, we found that the changes in the arrival times are not statistically significant, in the case of biologically relevant crowding levels (between 10 and 50% of the DNA being covered by DNA binding molecules), for both mobile and immobile obstacles.

Finally, we also measured the time the *O*_1_ site was occupied by a lacI molecule during one hypothetical *E.coli* cell cycle. The results show that, in the case of obstacles moving on the DNA, crowding decreases the average target site occupancy time (and this is statistically significant), while simultaneously the variation in occupancy is significantly increased. This means that noise can, in part, be accounted by the inherent crowding of molecules on the DNA and is supported by recent experimental evidence that non-cognate TFs contribute to gene expression noise (Sasson et al., 2012). In the case of fixed obstacles, we found the opposite effect, namely that increasing the crowding always leads to a statistically significant increase in the occupancy of the target site, but at the same time it also leads to a higher probability that the target site is never reached within the cell cycle. This suggests that in the case of fixed obstacles on the DNA, higher crowding can lead to a binary behavior of the occupancy of the target site (the target sites are occupied in fewer cells, but when they are occupied, they can display significant increase in occupancy time).

## 2. Results

### 2.1. Time to locate the target site

**Figure 1:**
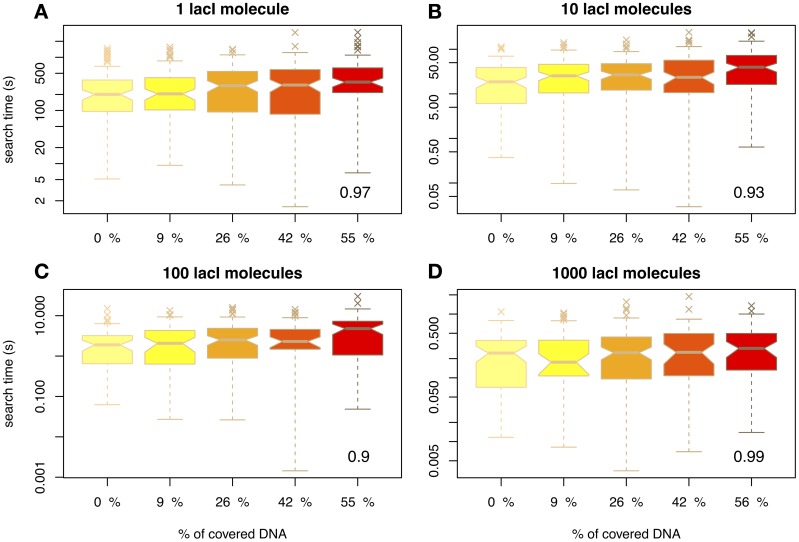
**The average time for the TF to reach the target site (measured in seconds) as a function of DNA crowding in the case of mobile obstacles.** Note the differences between scales of the y-axis, e.g. for 1 lacI molecule it takes in the range of tens of minutes to locate the target site, while for 100 copies the search time is in the range of seconds. The number in the inset represents the Pearson coefficient of correlation between crowding and the mean of the search time. The values indicate the crowding is highly correlated with the search time, in the sense that higher crowding on the DNA leads to higher search times. We used notched boxplots to represent the data, where: (1) the line in the box represents the median, (2) 50% of the data (the interquartile range *IQR*) occurs between the lower edge of the box (first quartile *Q*1) and upper edge of the box (third quartile *Q*3), (3) the lower whisker marks the maximum between (*Q*1 − 1.5 × *IQR*) and the lowest point in the data set, (4) the upper whisker marks the minimum between (*Q*3 + 1.5 × *IQR*) and the highest point in the data set, (5) the crosses represent the outliers and (6) the notches indicate the confidence intervals for the medians (if the notches of two medians do not overlap, the medians are significantly different at a 95% confidence level). Note that to enhance the visibility, the boxplots are positioned equidistant although the crowding levels are not. We considered four cases with respect to the number of lacI molecules, namely: **(A)** 1 molecule, **(B)** 10 molecules, **(C)** 100 molecules and **(D)** 1000 molecules.

First, we wanted to understand how crowding influences the association rate of a TF to its target site. Figure 1 shows the arrival times of the first lacI molecule to the *O*_1_ site for various abundances of non-cognate TFs and lacI. Figure 1A considers the case of 1 lacI molecule in the cell and several levels of crowding on the DNA and shows that, by increasing the amount of crowding, the mean arrival times always increase, but there is negligible change in the variance of the search time in the range of biologically relevant crowding levels on the DNA.

Nevertheless, we found that the increase in the search time is not statistically significant. In particular, we performed a Tukey's range test (for a 95% confidence interval) in conjunction with a One-Way ANOVA, which revealed that only in the case of 1 or 10 molecules of lacI, and crowding levels of at least 55% on the DNA, there is a statistically significant difference in the search time; see Figure S2 in the Supplementary Material. This result suggests that, for crowding within biologically relevant levels (between 10 and 50% of the DNA being covered by DNA binding proteins), the molecular crowding on the DNA has negligible effects. Li et al. (2009), found similar results, in the sense that they observed a low increase in the search time for crowding levels within biologically plausible levels. Nevertheless, since they performed an analytical study, they were able to identify only the mean search time, while here we show that when variability in the arrival time is included in the analysis, the increase in the search time become negligible.

Next, we wanted to confirm that the results of our simulations were in accordance with previous experimental studies. For example, Elf et al. (2007) found that the time of 1 lacI molecule to locate the *O*_1_ site is ≈ 354 s. For 10 molecules of lacI (which is the endogenous level of lacI in *E.coli*) the search time will be ten times faster, ≈ 35 s. Figure 1B shows that in our simulations 10 lacI molecules can locate the *O*_1_ site on average within similar times, but only for a degree of crowding levels of: 9% (〈*T*^0.09^〉 = 35.84 s), 26% (〈*T*^0.26^〉 = 35.52 s) and (〈*T*^0.42^〉 = 38.13 s). If there is no competition on the DNA, the time is shorter (〈*T*^0^〉 = 27.02 s), while for higher levels of crowding the time is higher (〈*T*^0.55^〉 = 52.05 s). This confirms that the system was correctly parameterized and that for biologically plausible crowding levels we obtain similar results to the experimental measurements. We can conclude that the arrival time for all considered crowding levels deviates only negligibly from the experimentally measured value; see also Figure S2 in the Supplementary Material. Furthermore, in the case of empty DNA, the mean search time is similar to the one proposed by Bauer and Metzler (2013), when they considered empty DNA and the 3D organization of the *E.coli* genome. This suggests that the 3D organization of the *E.coli* genome has only a limited effect on the search time.

**Figure 2:**
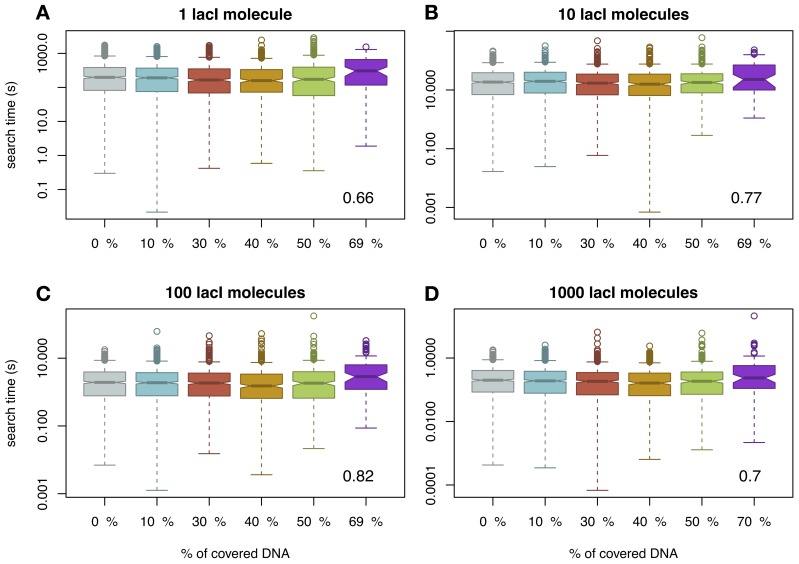
**The average time for the TF to reach the target site (measured in seconds) as a function of DNA crowding in the case of immobile obstacles.** For each set of parameters, we performed 1000 simulations. Note that the amount of covered DNA is higher than in the case of mobile obstacles, due to the fact that the molecules spend more time bound to the DNA. It should be noted that in the case of 70% of the DNA being covered by DNA binding proteins, the probability to locate the target site within a cell cycle is as low as 0.1; see Figure S1 in the Supplementary Material. The number in the inset represents the Pearson coefficient of correlation between crowding and the mean of the search time. Note that to enhance the visibility, the boxplots are positioned equidistant although the crowding levels are not. We considered four cases with respect to the number of lacI molecules, namely: **(A)** 1 molecule, **(B)** 10 molecules, **(C)** 100 molecules and **(D)** 1000 molecules.

One difference between our model and previous models (Li et al., 2009; Murugan, 2010) is that we assumed mobile obstacles, while the previous models assumed immobile obstacles. To investigate the impact of this assumption, we also performed a series of simulations where we considered the non-cognate TFs to be immobile obstacles as in Li et al. (2009). The description of this “TF species” can be found in the Materials and Methods section. In the case of immobile obstacles on the DNA, there is a different functional relationship between crowding on the DNA and the amount of time required by a TF to bind to its target site, in the sense that there is a crowding level on the DNA (or an interval of crowding) that minimizes the mean of the search time, thus, supporting the findings of Murugan (2010); see Figure 2. Although visually difficult to notice, we found that, in the case of 40% of the DNA being covered by immobile obstacles, there is a minimum in the mean of the search time. For example, if one lacI molecule needs on average 282 s to locate its target site in the case of naked DNA, then, in the case of 40% of the DNA being covered by immobile non-cognate molecules, the mean search time reduces to 244 s. Increasing the crowding level above this value leads to an increase in the search time up to 417 s (in the case of 70% of the DNA being covered by immobile obstacles). In *E.coli*, there seem to be ≈ 3 × 10^4^ molecules on the genomic DNA (Murugan, 2010), which potentially suggests that the abundance of DNA binding proteins in *E.coli* is set to minimize the search time of TFs for their target site. Nevertheless, these changes in search time are not statistically significant except for crowding levels of 70% (see Figure S3 in the Supplementary Material), which suggests that, for biologically relevant crowding levels on the DNA [between 10 and 50% (Flyvbjerg et al., 2006)], the search time is not significantly affected by the molecular crowding on the DNA or by the fact that the obstacles are mobile or immobile.

### 2.2. Proportion of time the target site is occupied

The second aspect we were interested in is the proportion of time the target site is occupied by cognate TFs, as this may have direct influence on gene expression. Sasson et al. (2012) found that binding sites of genes that are occupied by cognate TF molecules for shorter amounts of time display a larger degree of gene expression noise compared to binding sites that are occupied for longer times. They attributed this noise to the fact that cognate TF molecules can “insulate” the target site from non-cognate TF molecules. We wanted to verify the validity of this assumption and, thus, we measured the fraction of time the target site is occupied during stochastic simulation of the facilitated diffusion mechanism.

**Figure 3:**
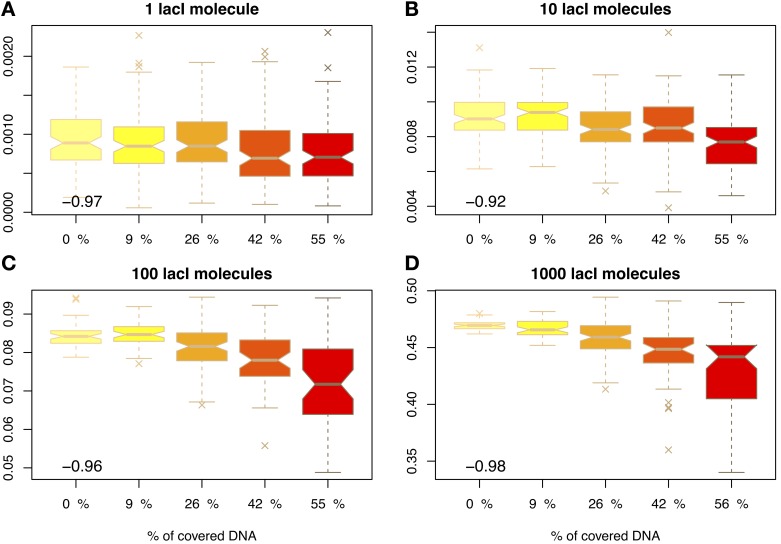
**Proportion of time relative to the cell cycle that the target site is occupied (y-axis) as a function of DNA crowding (x-axis) in the case of mobile obstacles.** The number in the inset represents the Pearson coefficient of correlation between crowding and the mean of the proportion of time the *O*_1_ site is occupied. The values indicate that crowding is highly anti-correlated with the proportion of time the target site is occupied, in the sense that higher crowding on the DNA leads to lower occupancy of the target site by cognate TFs. Note that to enhance the visibility, the boxplots are positioned equidistant although the crowding levels are not. We considered four cases with respect to the number of lacI molecules, namely: **(A)** 1 molecule, **(B)** 10 molecules, **(C)** 100 molecules and **(D)** 1000 molecules.

Figure 3 shows that molecular crowding on the DNA reduces the average occupancy of the target site, as previously proposed by Wasson and Hartemink (2009), and this reduction in occupancy is statistically significant (except in the case of 1 cognate molecule, which is usually attributed to leaky expression of the gene encoding the TF); see Figure S4 in the Supplementary Material. In the case of 10 molecules of lacI, the occupancy is reduced by 17% when the crowding increases from 9 to 55%. This means that crowding on the DNA can control gene expression levels at a global level. In the case of activating TFs, the increase in DNA-binding protein copy numbers may lead to a reduction in gene expression.

Furthermore, this reduction in the average occupancy also introduces a larger degree of variability that can be observed at target sites; see Figure 3. For example, in the case of 10 lacI molecules, the variance almost doubles (increase by 80%), when the crowding is increased from 9 to 55%. This higher variability, in conjunction with the lower occupancy of the target site, may result in an amplified increase of the noise in gene regulation; see Figure S7 in the Supplementary Material. One method to reduce the noise levels in the occupancy of the target site is increasing the abundance of the cognate TF (lacI in our case) (Becskei et al., 2005; Paulsson, 2005; Bar-Even et al., 2006; Zabet and Chu, 2010). Our results confirm that the increase in the noise levels generated by crowding can be compensated by an increase in lacI copy number.

**Figure 4:**
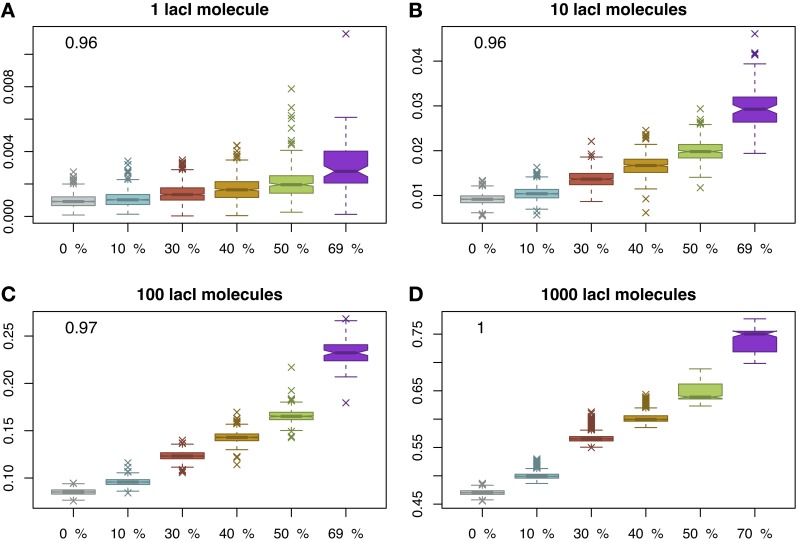
**Proportion of time relative to the cell cycle that the target site is occupied (y-axis) as a function of DNA crowding (x-axis) in the case of immobile obstacles.** For each set of parameters, we performed 1000 simulations. The mean occupancy of the target site is highly correlated with the crowding level on the DNA for all lacI abundances. Note that, for higher crowding, the number of simulations where the target site is reached within 3000 s decreases and, for 70% of the DNA being covered by DNA binding proteins, the probability to locate the target site drops to 0.1; see Figure S1 in the Supplementary Material. The number in the inset represents the Pearson coefficient of correlation between crowding and the mean of the proportion of time the *O*_1_ site is occupied. The values indicate that crowding is highly correlated with the proportion of time the target site is occupied, in the sense that higher crowding on the DNA leads to higher occupancy of the target site by cognate TFs. Note that to enhance the visibility, the boxplots are positioned equidistant although the crowding levels are not. We considered four cases with respect to the number of lacI molecules, namely: **(A)** 1 molecule, **(B)** 10 molecules, **(C)** 100 molecules and **(D)** 1000 molecules.

Finally, we considered again the case of immobile obstacles and measured the occupancy of the *O*_1_ site. Figure 4 displays an unexpected effect, namely that by increasing the crowding level, the occupancy of the target site increases as well and, again, this change is statistically significant; see Figure S5 in the Supplementary Material. The explanation for this result is that by increasing the molecular crowding on the DNA, the cognate molecules are confined more time in the vicinity of the target site as proposed by Wang et al. (2012). Nevertheless, in conjunction with this increase in occupancy of the target site, there is also a decrease in the number of simulations where the target site is reached. In other words, by increasing the crowding level on the DNA there are fewer cases where the target site is reached within one cell cycle (3000 s), but when (i.e., if) the target site is reached, the occupancy is higher, suggesting a change from a graded behavior (in the case of mobile obstacles) to a binary behavior (in the case of immobile obstacles).

## 3. Discussion

The influence that molecular crowding has on gene regulation has been considered only in a few previous studies. These studies mainly focused on the mean arrival time to the target site, such as Murugan (2010) and Li et al. (2009), or variability of target site occupancy (Sasson et al., 2012). Although these works provided analytical solutions on this issue, they did not consider the case of “mobile obstacles” on the DNA (Zabet and Adryan, 2012b). Here, we performed stochastic simulations where each molecule was explicitly represented, thus allowing an assessment of the difference between “mobile” and “fixed” obstacles.

Our results show that, in the case of immobile obstacles on the DNA, there is a crowding level that minimizes the mean of the search time (as found by Murugan, 2010), while, in the case of mobile obstacles, molecular crowding on the DNA (implemented through the presence of non-cognate TFs) increases the arrival time of cognate TFs to their target site (as previously proposed by Li et al., 2009). This increase in search time for high crowding levels on the DNA could potentially be explained by barriers forming in the vicinity of the target site as suggested in Ruusala and Crothers (1992), Hammar et al. (2012), and Wang et al. (2012). Nevertheless, we found that, within biologically relevant crowding levels, these changes in search time were small.

Recently, Marcovitz and Levy (2013) found similar results (for immobile obstacles there is a crowding level that minimizes the search time and for mobile obstacles the search time increases monotonically with the crowding levels), when they represented explicitly the 3D organization of the DNA and the 3D diffusion of the molecules. Note that this work studied the proportion of scanned nucleotides, which is related to the time required to locate the target site. However, they considered only 100 bp of DNA and obstacles that cover only 2 bp, which can introduce biases in the results if we consider real biological systems (Zabet, 2012). For example, in *E.coli*, molecules perform facilitated diffusion on ≈4.6 Mbp (genome-wide) (Riley et al., 2006) and they cover on average around 20 bp when bound to the DNA (Stormo and Fields, 1998). In contrast to Brackley et al. (2013), Marcovitz and Levy (2013) found that although the search time increases monotonically with rising crowding levels, this change is not significant. Their study considered 3D diffusion of molecules and a larger DNA fragment (≈4000 bp), only one molecule searching for its target site and the fact that crowding molecules would move slower on the DNA compared to the TF of interest.

Our results suggest that the change in arrival time, introduced by molecular crowding on the DNA, is not statistically significant for biologically plausible crowding levels in bacterial cells [in *E.coli*, between 10 and 50% of the DNA is covered by DNA binding proteins (Flyvbjerg et al., 2006)] irrespective of whether the obstacles on the DNA are mobile or fixed. Most importantly, these results are valid in the case of biologically derived parameters (affinities landscapes, multiple cognate TFs searching simultaneously, 4.6 Mbp of DNA, and between 10 and 50% molecular crowding). From this, one can conclude that the TF search time in bacterial cells is robust to changes in the molecular crowding level on the DNA. This result has a twofold implication: (1) for biologically relevant crowding levels on the DNA, the search time is not significantly affected by molecular crowding and (2) there is no statistically significant difference between fixed and mobile obstacles on the DNA with respect to the search time for biologically relevant crowding levels.

Importantly and in contrast to the search time, in the case of mobile obstacles on the DNA, crowding leads to a reduction in the proportion of time the target site is occupied and this reduction in occupancy is statistically significant. This may be an important feedback mechanism in cases where genes encode TFs. For example, in the case of activator TFs, an increase in activator TFs abundance will lead to an increase in crowding, which, consequently, results in a reduction of the binding of activator TFs to their target sites (thus, resulting in negative feedback). Analogously, in the case of repressing TFs, if the repression is achieved by blocking the binding of RNA polymerase to promoters, then an increase in crowding on the DNA would lead to further repression (again, resulting in negative feedback).

Genetic research and synthetic biology often employ experiments where the abundances of one or several TFs are changed significantly (either completely knocked down or significantly over-expressed). The general assumption is that only the genes that are directly regulated by the corresponding TFs (and to some extent their downstream targets) will be affected by this change. Nevertheless, significant changes in the overall abundance of DNA-binding proteins can lead to changes of the crowding on the DNA. Our study suggests that, in that case, the activity state of all genes can be affected by the changed degree of crowding. It can be assumed that evolution has come up with compensatory mechanisms that guarantee stable genomic expression levels, or that the degree of crowding must change significantly (beyond what is biologically feasible) for these effects to be measurable. This is where stochastic simulations can only inform us of theoretical possibilities, but where ultimately biological experiments are required.

In the case of immobile obstacles, crowding leads to a statistically significant increase in the occupancy of the target site, but, at the same time, the proportion of simulations where the target site is reached within a cell cycle drops significantly (mainly due to total or partial blockage of the target site by immobile obstacles). A bioinformatics study performed by Hermsen et al. (2006) revealed that, in *E.coli*, TF binding sites often overlap (they found that 39% of the binding sites overlap at least once) and this indicates that the exclusion of TFs from their target sites by molecular crowding on the DNA is a biologically plausible scenario. These two opposite effects suggest that, in the case of high number of fixed obstacles on the DNA, within the population the occupancy of the target sites display binary response, in the sense that in a subset of “virtual” cells the target site is never reached, but, in the rest of the “virtual” cells, the occupancy of the target site is greatly increased mainly due to the confinement of the TF molecule in the vicinity of the target site (Wang et al., 2012).

In both cases (mobile and immobile obstacles), crowding causes an increase in variability of the occupancy state across the population; see Figures S7, S8 in the Supplementary Material. Note that the variability here refers to population level variability and not time fluctuations, i.e., each simulation considers an independent “virtual” cell. This means that a cell that has a lower number of DNA-binding proteins may display a finer control on gene regulation and less gene regulation noise. In order to get more local control on gene regulation, lower crowding on the DNA is required, but crowding on the DNA in unavoidable. Hence, when the cell grows too much (in the sense of overall protein production) and the DNA gets overcrowded, the noise in gene regulation reduces the fitness of the cell, an aspect which can be compensated only if the cognate TF abundance increases as well. This indicates that when the cognate TFs are a fixed percentage of the total abundance of DNA-binding proteins, there is an optimal level of crowding above which the noise in gene regulation becomes harmful for the cell; similar to the results of Li et al. (2009).

Often it is assumed that there is a direct relationship between binding site occupancy and expression level. We show that the variability in occupancy is not negligible and depends on the number of non-cognate molecules bound to the DNA. This variability that can be observed between cells, is independent of fluctuations in the TF abundances (cognate or non-cognate), but arises from the facilitated diffusion mechanism and depends on crowding. In contrast, Bauer and Metzler (2013) found negligible variability in the search time, when they modeled the facilitated diffusion process assuming the 3D organization of the *E.coli* genome, but discarding the affinity landscapes of the TF. Here we show that the search time displays high variability when considering the TF affinity landscape, but this variability is not influenced significantly by the crowding levels on the DNA (in the case of mobile obstacles on the DNA); see Figure 1. In this context, the omission of variations in occupancy of the *cis*-regulatory region or wrong assumptions about its extent can generate misleading results when investigating the sources of noise in gene expression.

Overall, we found that only for immobile obstacles the occupancy of the target site is significantly higher (while the search time is only negligibly affected within biologically relevant levels of molecular crowding on the DNA, for both mobile and immobile obstacles); see Figure S6 in the Supplementary Material. This shows again how important the underlying assumption of immobile versus mobile obstacles is, in the case of genomic occupancy of TFs.

Slutsky and Mirny (2004) identified that the TF target search process is affected by the so-called speed-stability paradox, where the search process can be fast and lead to weak binding to the target site, or the search process can be slow and lead to strong binding to the target site. In the case of immobile obstacles, we showed that high crowding levels (which are within biologically plausible values) lead to higher occupancy at the target site and at the same the search time is not significantly affected. This suggests that the presence of immobile obstacles can potentially reduce the effects of the speed-stability paradox.

In this context, one might ask whether highly abundant fixed obstacles on the DNA really exist? In bacterial cells, given the high specificity of some TFs, we expect that a subset of the TFs would potentially create these immobile obstacles (e.g., CRP). However, given the low abundance of most other bacterial TFs (Wunderlich and Mirny, 2009), the position where these immobile obstacles emerge is encoded into the DNA. Thus, we cannot make a general statement regarding the molecular crowding on the DNA, but suggest this needs more systematic analysis for each particular promoter region.

Alternatively, barriers can form on the DNA when there is strong direct TF–TF cooperativity, which will lead to cluster formation on the DNA (Chu et al., 2009). This effect is removed when non-cognate TFs (that do not display direct TF–TF cooperativity) are present in the cell, but it is always the case that molecules that do not display cooperativity will be bound to the DNA.

Finally, the presence of nucleosomes on the DNA could be responsible for these barriers, but this is particular only for eukaryotic systems and there is still no clear evidence in what form facilitated diffusion exists in eukaryotic cells (Vukojevic et al., 2010; Gehring, 2011); discussed in Zabet and Adryan (2012b).

## 4. Materials and methods

We performed stochastic simulations using a computational framework and a set of parameters presented in Zabet and Adryan (2012a,c). [The previously published software (Zabet and Adryan, 2012c) and the manual can be downloaded from http://logic.sysbiol.cam.ac.uk/grip/download.html]. Briefly, the model represents explicitly all molecules in the system and allows to perform event driven stochastic simulations of the dynamics of the molecules in the system (Gillespie, 1976, 1977). The 3D diffusion is modeled implicitly by using the Master Equation, which was shown to be an accurate approximation when simulating binding of TFs molecules to the DNA (van Zon et al., 2006). The molecular crowding in the cytoplasm only scales the binding equilibrium constant and the 3D diffusion constant (Morelli et al., 2011). Our method to estimate the association rate to the DNA (Zabet and Adryan, 2012a) ensures that the TF molecules are bound to the DNA approximately 90% of the time, as it was experimentally measured in Elf et al. (2007), and this means that the effects of crowding in cytoplasm are implicitly incorporated in our model (through the association rate to the DNA).

Furthermore, our model assumes that the DNA is a string of letters {A,C,G,T} and, thus, we disregard the 3D organization of the *E.coli* genome. This aspect, the 3D organization of the genome, could potentially influence the search time as shown in Brackley et al. (2012) and Foffano et al. (2012). Bauer and Metzler (2013) considered a coarse grained model of the 3D structure of the *E.coli* genome and found that in the case of 1 TF molecule and empty DNA the mean search time is approximately 311 s. This value is similar to our result for empty DNA and 1 molecule of lacI searching on the DNA (282 s) and, thus, it seems that including the 3D organization of the *E.coli* genome would lead to only small deviations from our results.

The amount of time a molecule spends at a certain position on the DNA is a random number exponentially distributed with an average which is determined based on the binding energy (Gerland et al., 2002), here, approximated by the position weight matrix (Stormo, 2000). Once the amount of time spent at one position expires, the molecule can slide to a nearby position, hop on the DNA or unbind from the DNA with certain probabilities which were previously estimated in Zabet and Adryan (2012a). Finally, steric hindrance is implemented by not allowing two molecules to cover the same base pair simultaneously (Hermsen et al., 2006). In our system, we assume the existence of two TF species: a cognate (lac repressor in our case) and a non-cognate. The parameters associated with the lac repressor are listed in Table S1 in the Supplementary Material and its specificity expressed as position weight matrix in Table S2 in the Supplementary Material.

### 4.1. System size reduction

In Zabet (2012) we showed that it is sufficient to simulate the target finding process using a smaller (of at least 100 Kbp) region of DNA, provided that the parameters of the subsystem are adequately scaled. In particular, we found that there are two methods (the copy number model and the association rate model), which can be applied to adjust the parameters and that the copy number model can be used for highly abundant TFs (such as the non-cognate TFs in this case), while the association rate model for lower abundant TFs (lacI in this case).

To simulate non-cognate crowding we considered the following abundances for these TFs: (1) 0, (2) 10^4^, (3) 3 × 10^4^, (4) 5 × 10^4^, and (5) 7 × 10^4^ molecules. The association rate was set to the values listed in Table 1. This abundance of non-cognate TFs, the corresponding association rates and the fact that each molecules covers 46 bp of DNA lead to various percentages of DNA being covered, which reside in the range of biologically plausible values of 10–50% (Flyvbjerg et al., 2006) (except in the case of TF_nc_ = 0); see Table 1.

**Table 1:** **Sub-system parameters for various non-cognate molecule abundances**.

**TF_nc_**	***k*^assoc^_nc_ s^−1^**	**Covered DNA (%)**	**TF_nc_**	***k*^assoc^_1_lacI s^−1^**	***k*^assoc^_10_lacI s^−1^**	***k*^assoc^_100_lacI s^−1^**	***k*^assoc^_1000_lacI s^−1^**
0	1800	0	0	4.19	4.04	4.11	4.19
10,000	2000	9	216	4.58	4.63	4.67	4.74
30,000	2571	26	647	6.11	6.10	6.19	6.32
50,000	3600	42	1078	8.63	8.76	8.73	8.88
70,000	6000	55	1509	13.15	13.05	13.06	13.26

*The overbar is used to denote the corresponding parameters in the subsystem, e.g., TF_nc_ represents the abundance of non-cognate TFs in the 100 Kbp subsystem*.

For each set of parameters, we performed 50 simulations, each running for 3000 s, which is approximately the *E.coli* cell cycle (Rosenfeld et al., 2005). To increase simulation speed, we selected a 100 Kbp region of DNA which contained the *O*_1_ site (nucleotides 300,000–400,000 in the *E.coli* K-12 genome) (Riley et al., 2006). Since the non-cognate TFs are highly abundant, we applied the copy number model and obtained the corresponding abundances of non-cognate TFs (TF_nc_) for use in the subsystem, as listed in Table 1.

In addition to non-cognate TFs, the system also consists of cognate lacI molecules. We considered several lacI abundances: (1) 1, (2) 10, (3) 100, and (4) 1000 molecules. As well as in the case of non-cognate TFs, we used the same parameters for lacI as in previous work (Zabet, 2012; Zabet and Adryan, 2012a). In the case of the full system we considered an association rate of *k*^assoc^_lacI_ = 2400 s^−1^. When we applied the association rate model to reduce the system to 100 Kbp, we obtained the values of the association rate corresponding to each of the cases listed in Table 1.

### 4.2. Immobile obstacles

We also considered the case of *immobile* non-cognate molecules. These molecules are bound to the DNA at a random position (Berg et al., 1981) when the simulations start, and stay at that position until the simulations end. We allow immobile non-cognate TFs to cover the *O*_1_ site and, due to the fact that the model implements steric hindrace, the binding of any immobile non-cognate molecule within *T*^size^_lacI_ + *T*^size^_nc_ − 1 = 66 bp around the *O*_1_ site would exclude lacI molecules indefinitely from the *O*_1_ site.

For immobile obstacles, we performed 1000 simulations for each set of parameters and simulations where the target site is never reached are discarded. We found that, in the most extreme cases, (70,000 immobile non-cognate molecules) only 10% of the simulations lead to the target site being occupied by lacI within 3000 s; see Figure S1 in the Supplementary Material. Note that the number of simulations in the case of immobile obstacles is significantly higher compared to the mobile obstacles case. The main reason for that is that the simulation time is significantly shorter in the case of immobile obstacles compared to the case of mobile obstacles; i.e., in the case of immobile obstacles a simulation of 3000 s takes in the orders of hours, while, in the case of mobile obstacles, a simulation takes in the order of several weeks.

In the case of immobile obstacles, we also consider the case of 40,000 copies of non-cognate TFs. This was justified by the fact that, in the case of immobile obstacles, due to high residence time of the non-cognate TF molecules to the DNA, the percentage of DNA covered by molecules was higher than in the case of mobile obstacles. For 40,000 copies of non-cognate immobile molecules, 40% of the DNA was covered by DNA binding molecules, which is similar to the crowding level observed in the case of 50,000 copies of mobile non-cognate molecules. When we applied the copy number model and the association rate model to reduce the system to 100 Kbp, we obtained the following values: (1) TF_nc_ = 863 and (2) *k*^assoc^_1lacI_ = *k*^assoc^_10lacI_ = *k*^assoc^_100lacI_ = *k*^assoc^_1000lacI_ = 7.37. Note that in the case of immobile obstacles, the association rate affects the results negligibly as long as the binding to the DNA is fast compared to the amount of time spent bound to the DNA.

### Conflict of interest statement

The authors declare that the research was conducted in the absence of any commercial or financial relationships that could be construed as a potential conflict of interest.

## Supplementary material

The Supplementary Material contains: (1) the full list of parameters for the stochastic simulations, (2) the search time for immobile obstacles, (3) statistical test of the changes in the search time and occupancy between various levels of crowding, and (4) noise in the occupancy of the target site.
