# Supplementary material for: The effects of transcription factor competition on gene regulation
Source: Front Genet. 2013 Oct 7;4:197. doi: 10.3389/fgene.2013.00197 (PMC3791378; doi:10.3389/fgene.2013.00197)
Supplement: Supplementary file 1 [file DataSheet1.PDF]

Supplementary Material to: The effects of transcription factor competition on gene regulation.

Nicolae Radu Zabet[1,2,*] and Boris Adryan[1,2,†]

[1] Cambridge Systems Biology Centre, University of Cambridge, Tennis Court Road, Cambridge CB2 1QR, UK;

[2] Department of Genetics, University of Cambridge, Downing Street, Cambridge CB2 3EH, UK

*Email: n.r.zabet@gen.cam.ac.uk †Email: ba255@cam.ac.uk

TF parameters

The default parameters used here were previously derived in [1] and [2] and are listed in Table S1. In order to compare our results to the ones of [3] and [4], we considered also the case of *immobile* non-cognate TF species which are bound to the DNA at random positions during the initialisation step of the simulation and stay in the same position throught the entire simulation.

The PWM of the lacI was presented in [2] and is also listed in Table S2.

The number of simulations where the target site was reached

When a molecule binds to the DNA, it is uniformly distributed between all available position [5, 1]. This means that in the case of immobile obstacles, when the non-cognate molecules get bound to the DNA, there is a probability that they will bind to the target site (O_1) and, thus, in those simulations the target site is unreachable. We removed these points from the data and found that by increasing the number of non-cognate molecules, the probability of covering the target site also increases. Figure S1 confirms that, by increasing the crowding on the DNA, the number of simulations that resulted in the binding of lacI to O_1 site within 3000 s decreases.

We found that the proportion of simulations that resulted in the location of the target site within 3000 s is approximately: (i) $p^{0.1}_{reached} = 0.85$, (ii) $p^{0.3}_{reached} = 0.59$, (iii) $p^{0.4}_{reached} = 0.44$, (iv) $p^{0.5}_{reached} = 0.33$ and (v) $p^{0.7}_{reached} = 0.1$ (where the superscript indicates the proportion of DNA that is covered by DNA binding molecules); see Figure S1.

Statistical significance of the change in the search time

Figure S2 and Figure S3 confirm that for crowding levels on the DNA between 10% and 50% there is no statistically significant difference in the search time.

Statistical significance of the change in the occupancy of the target site

Figure S4 and Figure S5 confirm that for biologically relevant crowding levels on the DNA, there is a statistically significant difference in the occupancy of the target site. Note that for 1 molecule of lacI and mobile obstacles, the crowding level does not significantly change the occupancy of the target site; see Figure S4(A).

**parameter**	**lacI**	**non-cognate**	**notation**
copy number	see main manuscript		TF_x
motif sequence	see Table S2	-	
energetic penalty for mismatch	1 K_BT	13 K_BT	ε_x^*
nucleotides covered on left	0 bp	23 bp	TF_x^{left}
nucleotides covered on right	0 bp	23 bp	TF_x^{right}
association rate to the DNA	see main manuscript		k_x^{assoc}
unbinding probability	0.001474111	0.001474111	P_x^{unbind}
probability to slide left	0.4992629	0.4992629	P_x^{left}
probability to slide right	0.4992629	0.4992629	P_x^{right}
probability to dissociate completely when unbinding	0.1675	0.1675	P_x^{jump}
time bound at the target site	$1.18E-6$ s	0.3314193 s	τ_x^0
the size of a step to left	1 bp	1 bp	
the size of a step to right	1 bp	1 bp	
variance of repositioning distance after a hop	1 bp	1 bp	σ_{hop}^2
the distance over which a hop becomes a jump	100 bp	100 bp	d_{jump}
the proportion of prebound molecules	0.0	0.9	
affinity landscape roughness	-	1.0 K_BT	

Table S1: *TF species default parameters*

	**PWM**			
**Position**	A	C	G	T
1	0.6200	−0.6900	0.1400	−0.6900
2	0.6200	−0.6900	0.1400	−0.6900
3	0.1600	0.1400	−0.6900	0.1800
4	0.1600	−0.6900	−0.6900	0.6200
5	−0.7000	−0.7000	0.9000	−0.7000
6	−0.6900	−0.6900	−0.6900	0.9300
7	0.0077	−0.0084	−0.0073	0.0083
8	0.0077	−0.0084	−0.0073	0.0083
9	0.0077	−0.0084	−0.0073	0.0083
10	0.0077	−0.0084	−0.0073	0.0083
11	0.0077	−0.0084	−0.0073	0.0083
12	0.0077	−0.0084	−0.0073	0.0083
13	0.0077	−0.0084	−0.0073	0.0083
14	0.0077	−0.0084	−0.0073	0.0083
15	0.0077	−0.0084	−0.0073	0.0083
16	0.6200	−0.6900	0.1400	−0.6900
17	−0.7000	0.9000	−0.7000	−0.7000
18	0.9300	−0.6900	−0.6900	−0.6900
19	0.9300	−0.6900	−0.6900	−0.6900
20	−0.6900	0.1400	−0.6900	0.6200
21	−0.6900	0.1400	−0.6900	0.6200

Table S2: lacI PWM

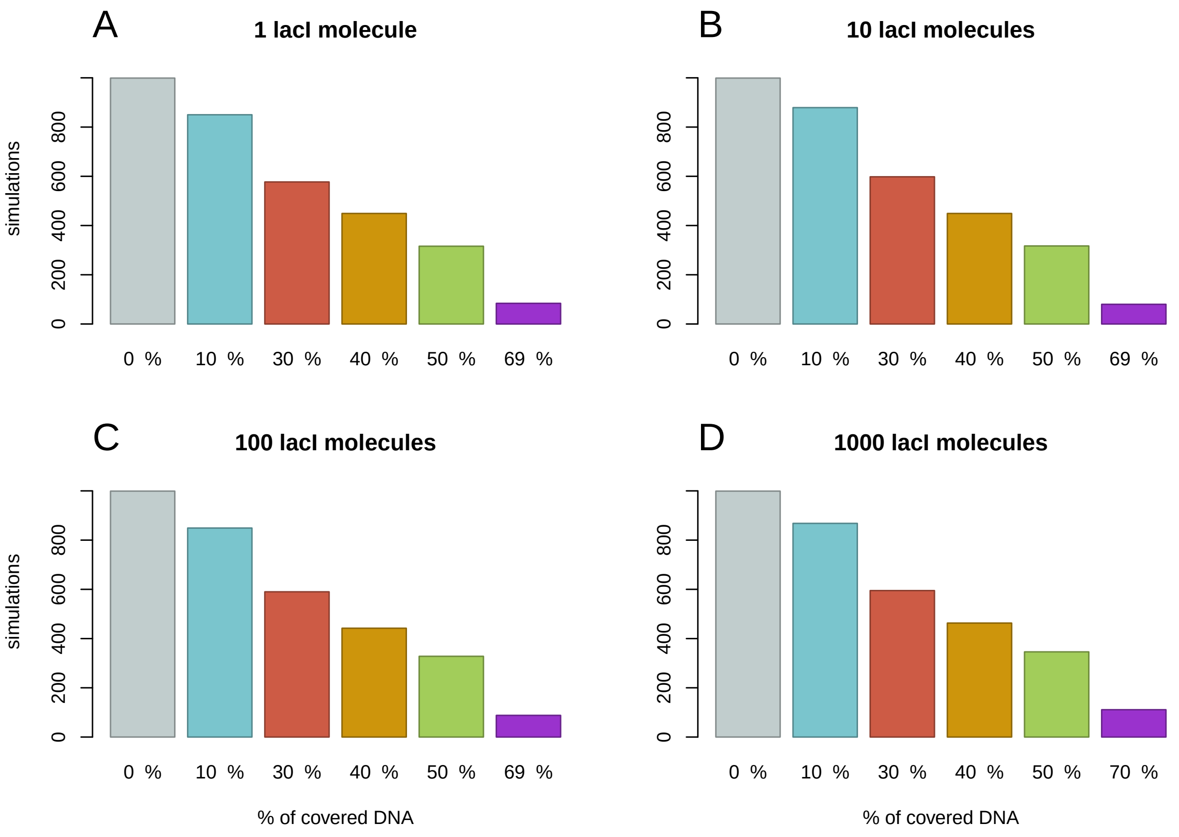

Figure S1: *The number of simulations where the target site was reached within* 3000 *s in the case of immobile obstacles.*

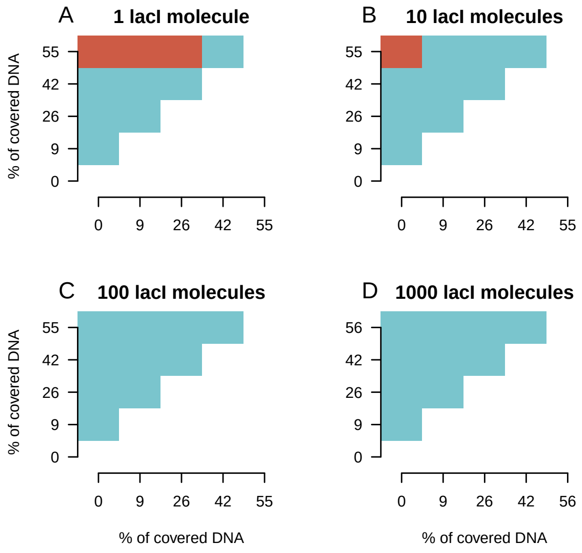

Figure S2: *Statistical significance of the change in the search time in the case of mobile obstacles.* The graph represents the pairwise statistical test between the distributions of arrival times to the target site at various crowding levels. We performed Tukey's range test (for a 95% confidence interval) on a one-way ANOVA of the logarithm of the search time. The color indicates the p-value of the difference between the corresponding search times. We represent by red the case of p-values lower than 0.05, and by blue the case of p-values higher than 0.05. The graph confirms that for crowding levels on the DNA between 10% and 50% there is no statistically significant difference in the arrival times to the target site.

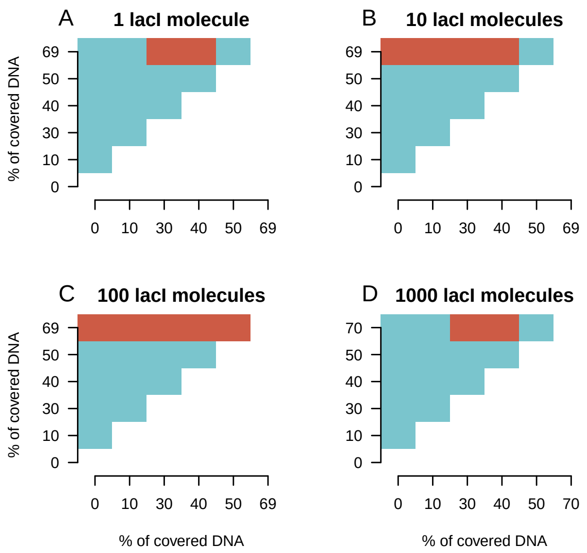

Figure S3: *Statistical significance of the change in the search time in the case of immobile obstacles.* The graph represents the pairwise statistical test between the distributions of arrival times to the target site at various crowding levels. We performed the Tukey's range test on the logarithm of the search time and the color indicates the p-value of the difference between the corresponding search times. We represent by red the case of p-values lower than 0.05 and by blue the case of p-values higher than 0.05. The graph confirms that for crowding levels on the DNA between 10% and 50% there is no statistically significant difference in the arrival times to the target site.

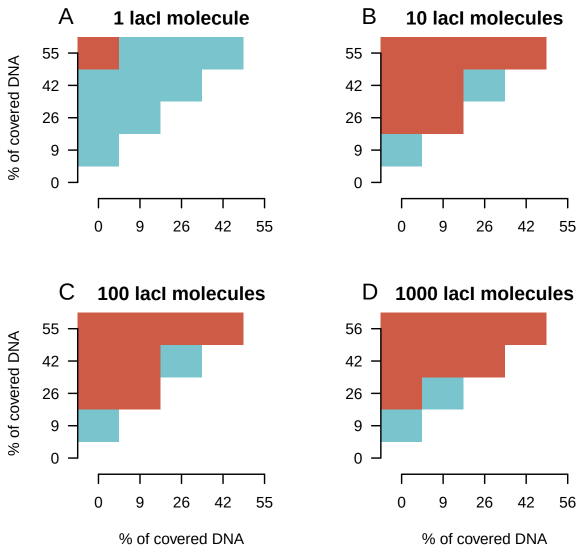

Figure S4: *Statistical significance of the change in the proportion of time the target site is occupied in the case of mobile obstacles.* The graph represents the pairwise statistical test between the distributions of occupancy of the target site at various crowding levels. We performed the Tukey's range test on the logarithm of the occupancy of the target site and the color indicates the p-value of the difference between the corresponding occupancies of the target sites. We represent by red the case of p-values lower than 0.05 and by blue the case of p-values higher than 0.05. The graph confirms that the crowding levels considered lead to statistically significant difference in the occupancy of the target site, except for the case of 1 lacI molecules (usually associated with leaky expression of the gene encoding the TF).

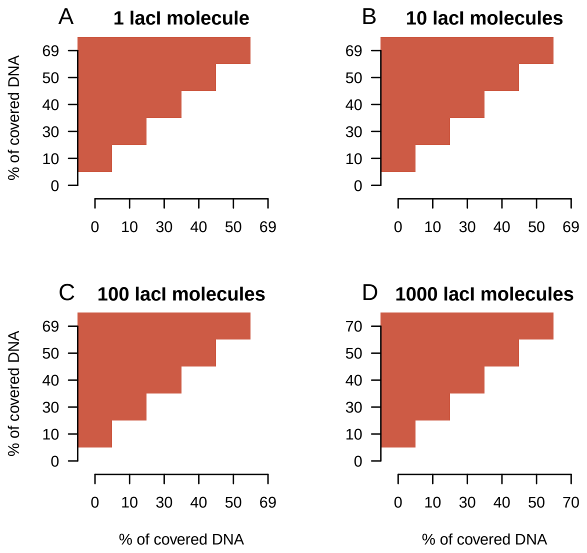

Figure S5: *Statistical significance of the change in the proportion of time the target site is occupied in the case of immobile obstacles.* The graph represents the pairwise statistical test between the distributions of occupancy of the target site at various crowding levels. We performed the Tukey's range test on the logarithm of the occupancy of the target site and the color indicates the p-value of the difference between the corresponding occupancies of the target sites. We represent by red the case of p-values lower than 0.05 and by blue the case of p-values higher than 0.05. The graph confirms that the crowding levels considered lead to statistically significant difference in the occupancy of the target site.

Comparison between the mobile and immobile obstacle case

Finally, we compared the overall mean occupancy of the target site between the case of mobile and immobile obstacles. Our results showed that, when the obstacles are fixed on the DNA, the occupancy of the target site is higher (see Figure S6).

Proportion of time the target site is occupied in the case of mobile obstacles

We also looked at the noise in occupancy and found that indeed, there is a strong correlation between crowding levels on the DNA and noise in the proportion of time the target site is occupied. In particular, we found that by increasing the level of crowding on the DNA the noise in the occupancy of the target site is increased; see Figure S7. Interestingly, this is valid for both mobile (Figure S7) and immobile obstacles (Figure S8).

References

[1] Nicolae Radu Zabet and Boris Adryan. A comprehensive computational model of facilitated diffusion in prokaryotes. *Bioinformatics*, 28(11):1517–1524, 2012.

[2] Nicolae Radu Zabet. System size reduction in stochastic simulations of the facilitated diffusion mechanism. *BMC Systems Biology*, 6(1):121, 2012.

[3] Gene-Wei Li, Otto G. Berg, and Johan Elf. Effects of macromolecular crowding and dna looping on gene regulation kinetics. *Nature Physics*, 5:294 – 297, 2009.

[4] R Murugan. Theory of site-specific interactions of the combinatorial transcription factors with dna. *Journal of Physics A: Mathematical and Theoretical*, 43:195003, 2010.

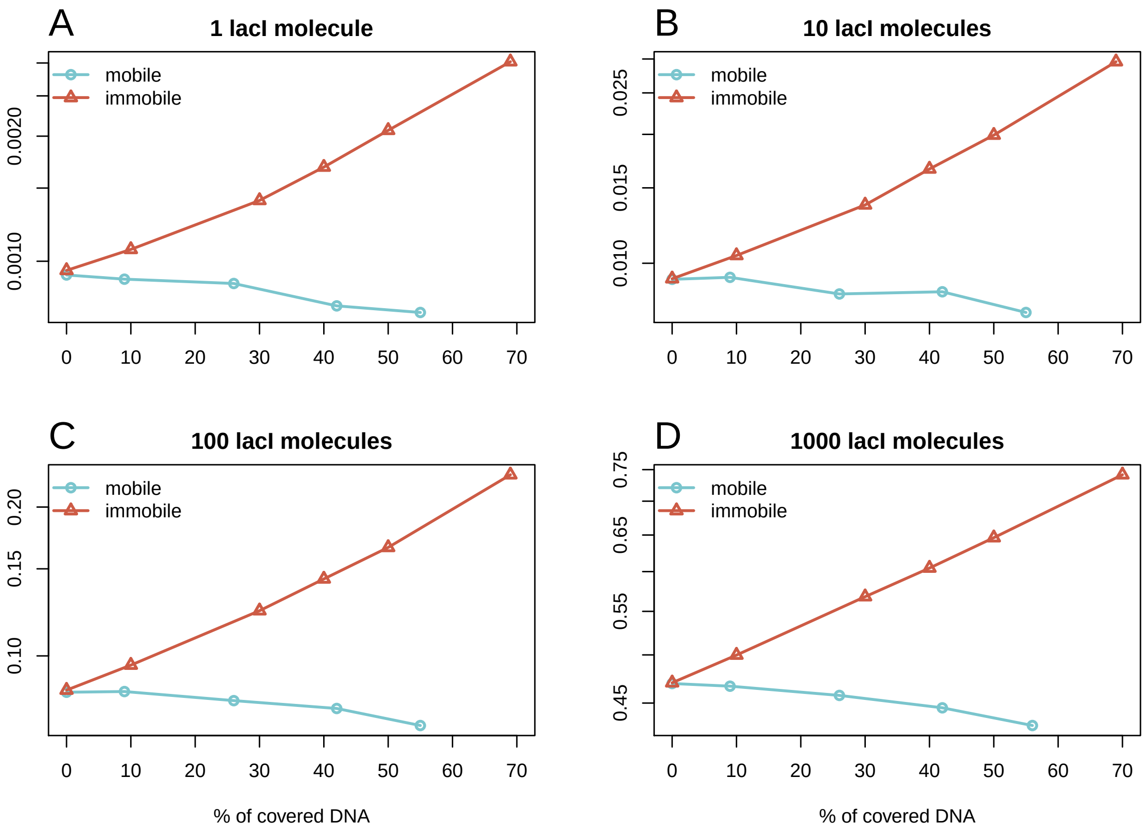

Figure S6: *The comparison between the mean occupancy time of the target site in the case of mobile obstacles and in the case of immobile obstacles.*

[5] Otto G. Berg, Robert B. Winter, and Peter H. von Hippel. Diffusion-driven mechanisms of protein translocation on nucleic acids. 1. models and theory. *Biochemistry*, 20(24):6929–6948, 1981.

[6] Johan Paulsson. Models of stochastic gene expression. *Physical Life Reviews*, 2:157–175, 2005.

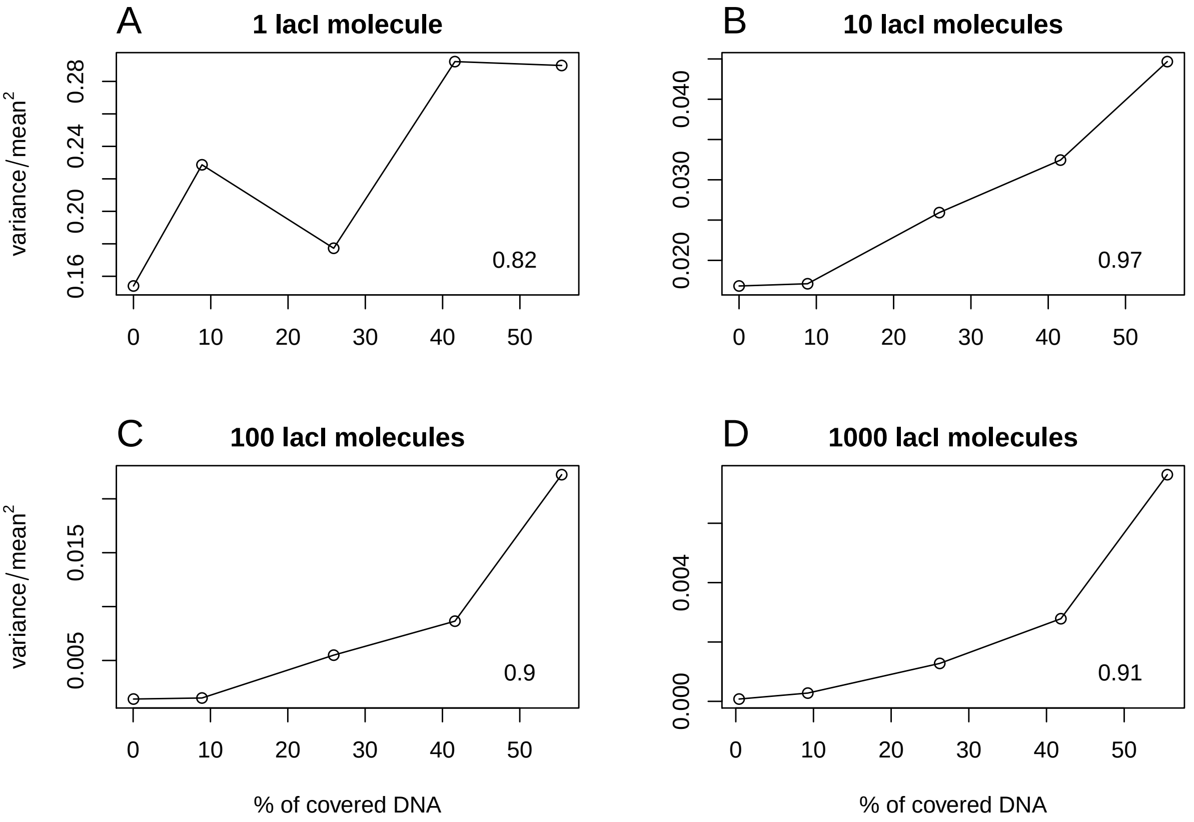

Figure S7: *Noise in the proportion of time the target site is occupied as a function of the crowding levels on the DNA in the case of mobile obstacles.* We normalised the variance by the square of the mean as proposed in [6]. The number in the inset represents the Pearson coefficient of correlation between crowding and the noise in the proportion of time the target site is occupied.

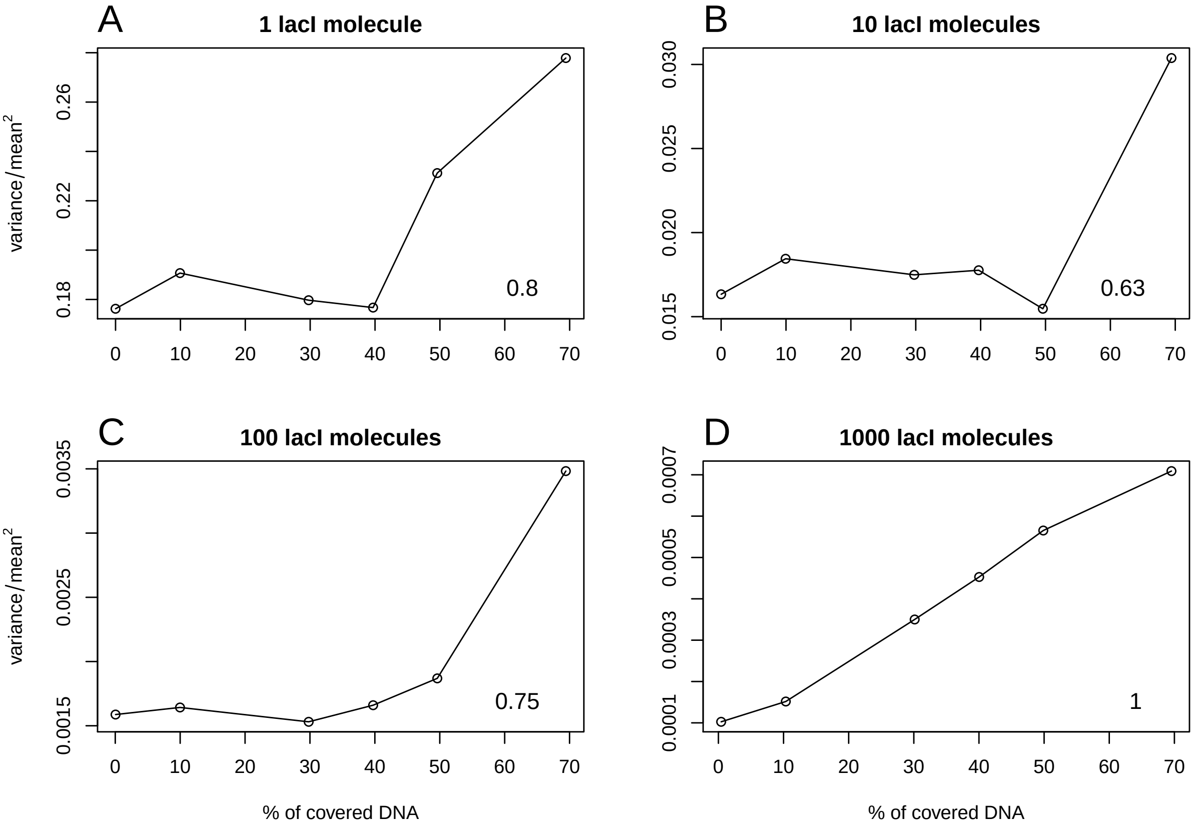

Figure S8: *Noise in the proportion of time the target site is occupied as a function of the crowding levels on the DNA in the case of immobile obstacles.* We normalised the variance by the square of the mean. The number in the inset represents the Pearson coefficient of correlation between crowding and the noise in the proportion of time the target site is occupied.
